# Complications of New Medications

**DOI:** 10.5811/westjem.2014.10.23837

**Published:** 2014-11-13

**Authors:** Carlos J. Roldan, Linda Paniagua

**Affiliations:** The University of Texas Health Science Center at Houston, Department of Emergency Medicine, Houston, Texas

## Abstract

Numerous mandibular pathologies are diagnosed in the emergency department (ED). We present the case of a woman with severe right-sided mandibular pain who was found to have a pathological fracture and osteonecrosis of the jaw (ONJ). The etiology of ONJ was found to be associated to previous use of zoledronic acid to treat osteoporosis. The aim of this case report is to discuss the etiology, diagnosis and treatment of ONJ secondary to the use of zoledronic acid and to outline a clinical condition rarely seen in the ED whose incidence might rise with the increasing use of bisphosphonates.

## INTRODUCTION

Osteonecrosis, the destruction and death of bone tissue, has traditionally been attributed to ischemia, infection, neoplastic disease and trauma. Reports in the medical literature have begun to establish a direct correlation with the use of pharmacological agents intended to prevent skeletal fractures in patients with malignancies such as multiple myeloma and prostate cancer.^1^ Among those pharmacological agents, bisphosphonates (BPs) have gained popularity in the non-cancer population for the treatment of postmenopausal osteoporosis. The intended clinical benefits are not risk free. Among other effects, BPs have been shown to stimulate the gamma-delta T cells, which can potentially contribute to indiscriminate cytotoxicity against tumor cells and normal cells.^2^ A wide range of additional systemic side effects have been described. Of particular concern is structural bone disease manifested as increased risk of fractures of the hip and pelvis and osteonecrosis of the mandible. It is unknown whether the risk continues after stopping the therapy. Localized pain is an early manifestation of bone compromise. In patients using BPs this is considered a warning sign of bone complications.

## CASE REPORT

A 73-year-old woman with history of hypertension and osteoporosis presented to a suburban emergency department (ED) complaining of right-sided mandibular pain and gum swelling for one week. She was diagnosed with gingivitis and discharged home on oral antibiotics, analgesics and a mouth rinse solution. She was advised to follow up with the dental service. After two weeks the symptoms continued to progress. She presented to our ED once the pain became unbearable after feeling a “pop” while trying to chew peanuts with the left side of her mouth. She denied malaise, fever, anorexia or any history of trauma. Our evaluation revealed a very uncomfortable-appearing female. She was afebrile (temperature 98.2°F) and had normal vital signs other than a blood pressure of 169/92mm Hg and heart rate 104 beats per minute. Her physical exam was remarkable for right mandibular edema, tenderness and crepitance to palpation. Her dentition was in fair condition and she had scattered fillings. A grayish discoloration of the gingiva was evident in the premolar zone with no apparent drainage. Laboratory values were within normal limits, including a white blood cell count of 8,000 cells/uL with 60% neutrophils. A computed tomography demonstrated “osteonecrosis of the jaw and a pathological fracture of the right ramus” (Figure). Further chart review established that the patient had been previously treated for osteoporosis with intravenous zoledronic acid once a year for two years. The most recent dose was one month earlier. The patient was hospitalized by ear nose throat surgery, and surgical reconstructive therapy was performed. Seven days later, her pain had nearly resolved and she was discharged home.

## DISCUSSION

In 1995, the U.S. Food and Drug Administration approved the first BP agent. Since then several oral and intravenous BPs have been released to the market. BPs are used to inhibit resorption of bone mass in patients with bone metastases, bone primary lesions and for the treatment of osteoporosis.^3^ With our aging population osteoporosis is becoming a matter of public health. This bone deficiency is estimated to result in two million fractures every year.^4^ The estimated annual expenditure is between $12.2 to 17.9 billion.^5^

Zoledronic acid, currently the most-prescribed intravenous BP for the treatment of osteoporosis, is also used for the treatment of hypercalcemia associated with malignancy, multiple myeloma and bone metastases from solid tumors. Despite being highly effective in reducing the risk of osteoporotic fractures, a number of safety concerns have surfaced. Atypical subtrochanteric and femoral fractures and osteonecrosis of the jaw have sparked debate over the safety issues associated with long-term use of these drugs.

Osteonecrosis of the jaw (ONJ), also known as avascular or aseptic necrosis of the mandible, jaw death, dead jaw disease, or bisphossy jaw, was first described by Marx et al. a decade ago. 6 It is a disfiguring and disabling condition under which the mandible bones suffer literal death. Although the exact pathophysiology of how ONJ relates to BP use has not been found, several pathogenic mechanisms have been proposed. The low bone turnover induced by BPs can lead to decreased blood flow, bone cell necrosis and apoptosis.^6^,^7^ In conjunction with infection, decreased blood flow can lead to the development of non-healing exposed bone in the mouth.^6^,^7^ Recent data suggest that mucosal damage is the event preceding infection and subsequent bone necrosis. A retrospective chart review of oncology patients (n= 4,000) treated with BPs suggested that mucosal damage was an important precipitating factor of ONJ.^8^ The higher incidence of ONJ in cancer patients might be explained by the higher frequency of concomitant immunosuppression and the use of anti-cancer drugs and corticosteroids. The increasing evidence in the literature tying BPs and infection together cannot be dismissed.^9^

It is important to mention that the high levels of nitrogen content of some BPs, zoledronic acid among them, have been tied to ONJ to a much higher degree than the less nitrogen containing BPs.^10^ In support of this, a review of 368 cases of BPs-cancer associated ONJ found that patients receiving intravenous nitrogen-containing BPs are at greatest risk for ONJ.^11^

In light of the current literature, it is imperative that clinicians be aware of the risk and benefits of BPs. There has been evidence suggesting that dental trauma, surgical procedures and extractions and increased dosage of BPs could increase the risk of ONJ.^12^,^13^ It is also presumed that diabetes, autoimmune conditions, periodontal disease, poor oral hygiene and poorly fitting dentures may increase the risk of developing ONJ.^14^ A close dental examination and preventive dentistry prior to beginning zoledronic acid is recommended. A stricter patient selection and education with close follow up might be a reasonable strategy to monitor the development of these complications.

The role of the emergency physician is to be aware of the clinical significance of localized bone pain, even in the absence of trauma, in patients under current or past therapy with BPs. Clinical suspicion should be followed by diagnostic imaging and proper referral for definitive treatment. The current treatment of ONJ belongs to the surgical specialties; although treatment protocols have been outlined, there has been no randomized clinical trial assessing management strategies. Thus, management of ONJ remains controversial.

## Figures and Tables

**Figure:**
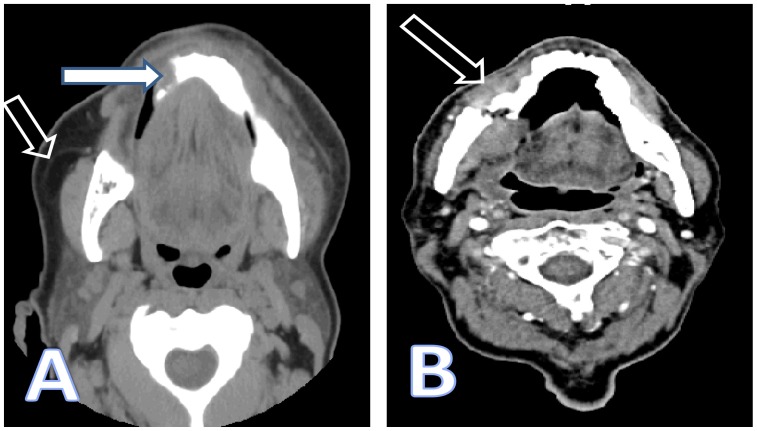
Computed tomography of the face without (A) and with (B) intravenous contrast. (A) Mandibular sclerosis with periosteal reaction of the mandibular body extending to the parasymphyseal region; given history of bisphosphonates therapy most likely osteonecrosis (full arrow). Diffuse subcutaneous edema and submental soft tissue swelling reflecting focal inflammatory changes (hollow arrow). (B) Osteoradionecrosis of the mandible with pathologic fracture of the right horizontal mandibular ramus (arrow).
